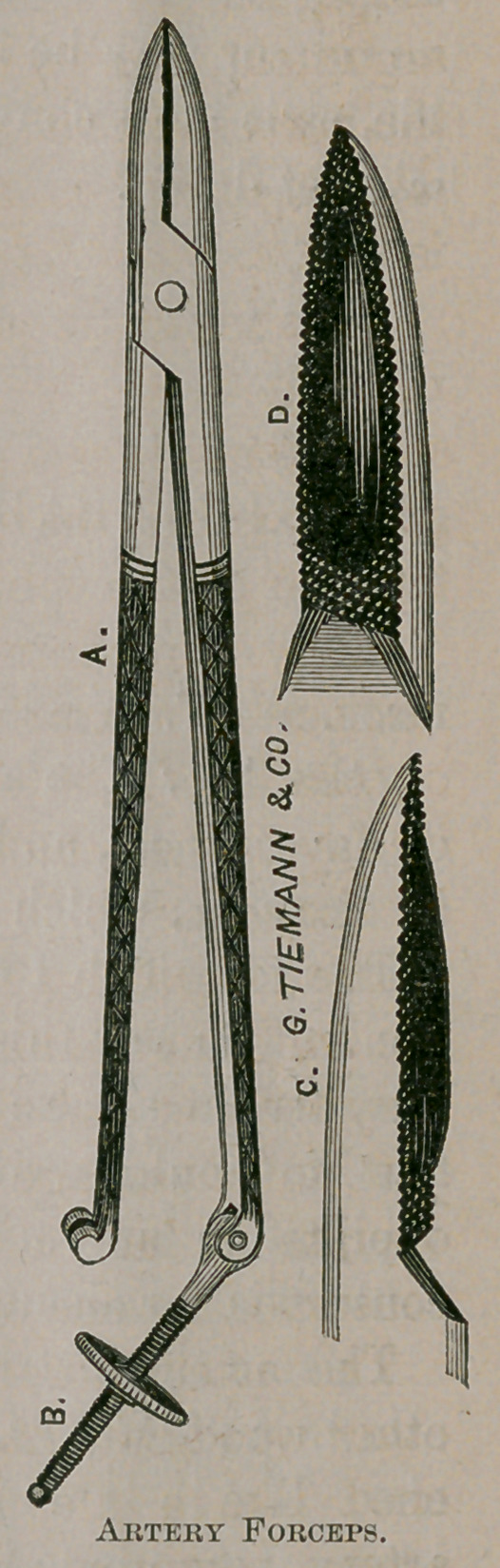# Recti-Linear Ecraseur—Artery Forceps

**Published:** 1871-10

**Authors:** J. C. Nott

**Affiliations:** New York


### RECTI-LINEAR ECRASEUR—ARTERY FORCEPS.

New York, October 10th, 1871.

Prof. W. F. Westmoreland:

*Dear Sir:* The accompanying wood cuts represent a *recti-linear ecraseur*, and an *artery forceps* upon the principle of an ecraseur, which have occupied my attention for some time past, and which I think worthy of the notice of the profession. You saw this *ecraseur* tested, during your late visit to this city, in a case of internal piles, and expressed a desire that I should write a little article for your journal on its application and uses. I now, though more hastily than I could wish, redeem my promise.

I name the instrument *recti-linear ecraseur*, because it operates in a *right line*, whereas that of Chassaignac cuts in a circle. My instrument has two straight blades, three inches long. One blade has a narrow fenestra its whole length, and the other a thin rough edge which passes into the opposite fenestra when the instrument is closed. The instrument is applied on a pile, or any part to be removed, at the point indicated. The blades are then brought together firmly by a screw in the ends of the levers, and the tissues are crushed down into a small pulpy mass, *without being severed.* If any

Recti-Linear Ecraseur.

Artery Forceps.

other part than a pile is operated upon, the projecting part is cut away with knife or scissors, leaving the crushed tissues *in situ*, the crushed vessels being allowed to remain. The protection against bleeding is much more perfect than where the tissues are cut entirely through by the instrument of Chassaignac. It is always better to allow the instrument to remain on a few minutes, in order to glue the tissues well together, and allow a little time for coagulation. In operating on piles, I always consider it safest to take the instrument off and then *apply a ligature in its track.* In this way, you guard against any possibility of hemorrhage, and the tissue being reduced by the ecraseur to a *soft pulp*, *a very small pedicle is left for the ligature*, *which cuts its way through in from* 24 *to* 48 *hours.* The part of the tumor projecting beyond the ligature, I usually snip off with scissors. The strongest argument, however, in favor of this *ecraseur*, is the fact that the nerves are so crushed and paralyzed that there is *no subsequent pain from the ligature*, as is always seen when the pile is tied, *without being preceded by the ecraseur.*

An anæsthetic should always be given when the instrument is used; and, *in all operations* for hemorrhoids, the sphincter ani, as a preliminary step, should be well dilated, to guard against the protrusion of the rectum and painful contraction of the sphincter upon it, after the operation.

Many directions are given in our surgical works on the manner of ligaturing piles; but, I do not care how it is done, or who does it, it is often followed by great suffering for hours, or days, when the ligature is applied without being preceded by the *ecraseur.*

The method by clamp and actual, or galvano-cautery avoids the suffering caused by simple ligature; but it is alarming, very tedious, and expensive, and, with all these disadvantages, has no advantage over the ecraseur, with ligature. I can operate on half a dozen cases, with my *ecraseur*, in the time consumed by one operation with the galvano-cautery.

The artery forceps, above alluded to, (represented in the other wood cut,) has the internal surfaces of the blades roughened like a file, and, when applied to a single bleeding artery, compress and dovetail the coats so together as to render it impervious to blood. The principle is, simply, that of *ecrasement*, which must act on *one* artery, by itself, as on several, at the same time, when a large instrument is used.

A hemorrhoid is a peculiar growth, and requires great caution to avoid hemorrhage. It is usually a bleeding tumor, pouring out blood from its *surface*. When removed by the *ecraseur* of Chassaignac, they sometimes bleed to an alarming, or even fatal, extent; because the vessels *around* the outer margin of the instrument are likely to be torn by the dragging action of the chain or wire, even if those within the loop should be perfectly closed. The same objection, I fear, holds against my instrument, and I have been afraid to trust it alone. I, therefore, in operating for piles, after crushing the pedicle well, with my ecraseur, remove the instrument and *tie a ligature firmly in its track*. The instrument, of course, must give pain if an anæsthetic be not used; but the nerves of the part are so deadened, that a ligature gives no acute pain. There is always a sensation of soreness after such operations.

When, however, the instrument is used to remove a tongue, penis, cervix uteri, portion of the vagina, etc., there is no need for ligatures, and the part projecting beyond the grip of the blades may be shaved off closely with knife or scissors, and, then, the instrument removed.

This instrument answers well, in the operation of ovariotomy, to sever adhesions between the tumor and walls of the abdomen, and, I think, might be trusted to divide the pedicle of an ovarian tumor—but here it has not been tested.

I think one of the most important uses of this ecraseur will be found in the operation of removing portions of the vagina, for procidentia. I have tested it in this operation, and have been much pleased with its action.

The instrument I have used answers well in all the cases I have tried it; but there may be modifications, perhaps, in size and shape, to suit other indications.

Very respectfully, your ob't serv't,

J. C. Nott, M.D.

## Figures and Tables

**Figure f1:**
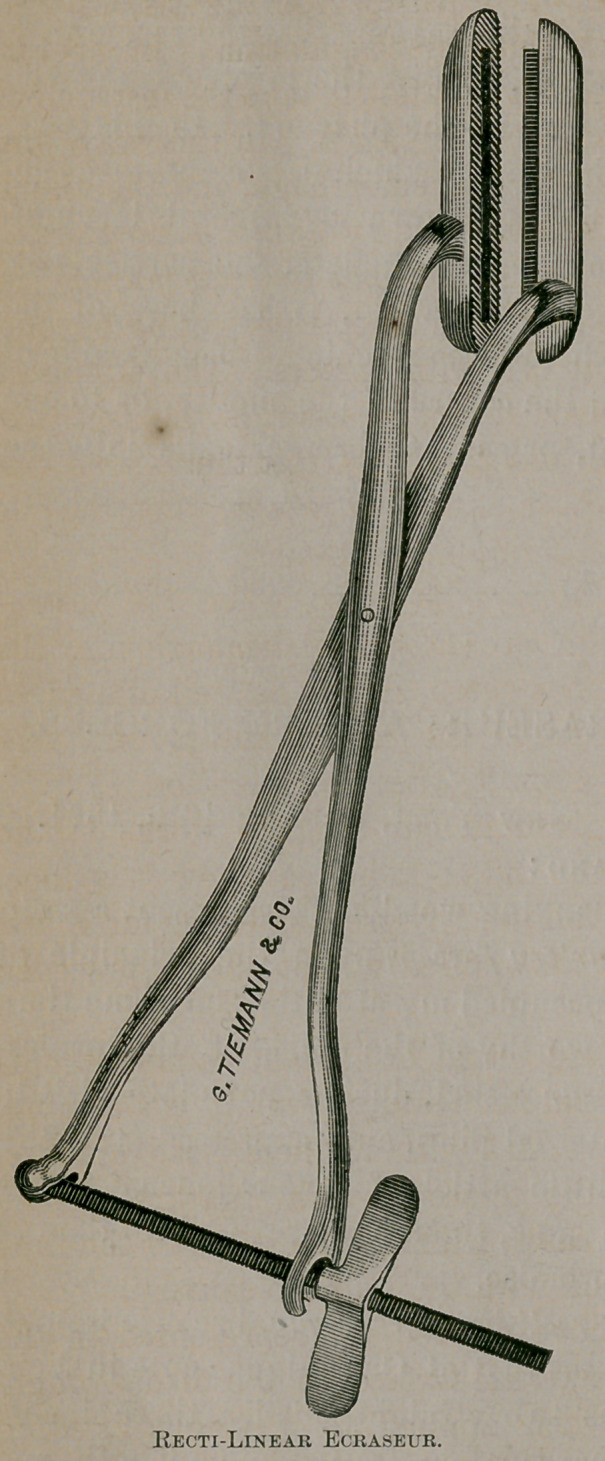


**Figure f2:**